# Genome Sequence of *Lysinibacillus sphaericus* Strain KCTC 3346^T^

**DOI:** 10.1128/genomeA.00625-13

**Published:** 2013-08-15

**Authors:** Haeyoung Jeong, Da-Eun Jeong, Young Mi Sim, Seung-Hwan Park, Soo-Keun Choi

**Affiliations:** Korean Bioinformation Center, Korea Research Institute of Bioscience and Biotechnology (KRIBB), Yuseong-gu, Daejeon, Republic of Koreaa; Super-Bacteria Research Center, KRIBB, Yuseong-gu, Daejeon, Republic of Koreab

## Abstract

*Lysinibacillus sphaericus* is a heterogeneous species that includes strains that produce mosquitocidal toxin proteins. Herein, we report the 4.56-Mb draft genome sequence of the nonpathogenic *L. sphaericus* strain KCTC 3346^T^, which provides clues for the phylogenetic reassessment of *L. sphaericus* species and an understanding of its physiological properties.

## GENOME ANNOUNCEMENT

*Lysinibacillus* was first proposed as a novel genus related to *Bacillus*, owing to its distinctive peptidoglycan composition, phylogenetic analyses, and physiology (1). *Lysinibacillus sphaericus* (formerly known as *Bacillus sphaericus*) and *Bacillus thuringiensis* subsp. *israelensis* produce insecticidal toxin proteins against mosquito larvae, and therefore, they have long been used for the biological control of vector species that transmit tropical diseases (2, 3). *L. sphaericus* strains are subdivided into five major groups (I to V) on the basis of their DNA homologies (4). Though these groups likely represent separate species, there are few phenotypic tests that are available to distinguish them. All mosquito-pathogenic strains belong to groups IIA and IIB (5), while the nonpathogenic strain KCTC 3346^T^ (=ATCC 14577 = DSM 28 = IAM 13420) belongs to group I.

There is currently only one available complete genome sequence of *L. sphaericus* (that of strain C3-41) that has significantly higher activity against *Culex* spp. than the commercialized strain *L. sphaericus* 2362 (6). In this study, we chose the type strain of *L. sphaericus* for sequencing to provide genomic information that is indispensable for understanding the relatedness of *L. sphaericus* strains and their characteristics as potent agents for biological control.

KCTC 3346^T^ was purchased from the Korean Collection for Type Cultures, and genome sequencing was carried out using an Illumina HiSeq 2000 system by the National Instrumentation Center for Environmental Management at Seoul National University (Seoul, Republic of Korea). Paired-end reads of 101 bp (3.33 Gb) produced from an ~450-bp genomic library were processed and *de novo* assembled using the CLC Genomics Workbench v6.0.4 (CLC bio). We obtained 75 scaffolds (≥179 bp) composed of 90 contigs (total contig length excluding scaffolded regions, 4,561,354 bp; 37.1% G+C content). The maximum contig length and N_50_ were 421,037 bp and 213,522 bp, respectively. No putative plasmid sequences were found in the assembly.

Automatic genome annotation was performed using the RAST server (7), which contains 4,499 protein-coding sequences and 86 tRNA genes, with 1,331 sequences (29.6%) annotated as hypothetical proteins. The closest neighbors suggested by the RAST server were *Bacillus* sp. NRRL B-14905 (8) and *L. sphaericus* C3-41 (6). However, the average nucleotide identity values between KCTC 3346^T^ and B-14095 or C3-41, as calculated using the JSpecies program (9), were as low as ca. 78.5%. Moreover, genes encoding insecticidal toxins, such as Mtx and BinA/BinB, could not be identified from the assembled sequence. A 16-kb nonribosomal peptide synthetase/polyketide synthase (NRPS/PKS) gene cluster was found in contig 6_2, but no antibiotic activity was demonstrated against *Rhizoctonia solani*, *Botrytis cinerea*, *Micrococcus luteus*, or *Escherichia coli*. Plant growth-promoting activity against *Arabidopsis thaliana* was also not shown.

In summary, our analysis results further support that the species *L. sphaericus* should be dissected into different groups, as the aforementioned DNA homology studies and other experimental evidences have suggested. The availability of their genome sequences will provide insight into the relationships and functionalities of *L. sphaericus* strains.

### Nucleotide sequence accession numbers.

This whole-genome shotgun project has been deposited at DDBJ/EMBL/GenBank under the accession no. AUOZ00000000. The version described in this paper is version AUOZ01000000.
